# Implementation of the Rank-Weighted Co-localization (RWC) algorithm in multiple image analysis platforms for quantitative analysis of microscopy images

**DOI:** 10.1186/s13029-016-0048-8

**Published:** 2016-02-16

**Authors:** Vasanth R. Singan, Jeremy C. Simpson

**Affiliations:** School of Biology and Environmental Science, Science Centre West, University College Dublin, Dublin 4, Ireland; DOE Joint Genome Institute, Walnut Creek, CA 94598 USA

## Abstract

**Background:**

Quantitative co-localization studies strengthen the analysis of fluorescence microscopy-based assays and are essential for illustrating and understanding many cellular processes and interactions. In our earlier study, we presented a rank-based intensity weighting scheme for the quantification of co-localization between structures in fluorescence microscopy images. This method, which uses a combined pixel co-occurrence and intensity correlation approach, is superior to conventional algorithms and provides a more accurate quantification of co-localization.

**Findings:**

In this brief report we provide the source code and implementation of the rank-weighted co-localization (RWC) algorithm in three (two open source and one proprietary) image analysis platforms. The RWC algorithm has been implemented as a plugin for ImageJ, a module for CellProfiler and an Acapella script for Columbus image analysis software tools.

**Conclusions:**

We have provided with a web resource from which users can download plugins and modules implementing the RWC algorithm in various commonly used image analysis platforms. The implementations have been designed for easy incorporation into existing tools in a ‘ready-for-use’ format. The resources can be accessed through the following web link: http://simpsonlab.pbworks.com/w/page/48541482/Bioinformatic_Tools.

## Introduction

The quantitative co-localization of markers in microscopy images has been widely used to study the spatial organization of intracellular environments. Traditional co-localization algorithms are based on either correlation of intensity values or pixel co-occurrence within regions of interest. We recently developed an algorithm that combines the information coming from intensity and pixel co-occurrence, and demonstrated in different scenarios that this rank-weighted co-localization (RWC) method produces superior results to traditional methods for quantification of co-localization [1]. While traditional co-localization methods that consider either pixel co-occurrence or intensity correlation have limitations, by integrating the two methods RWC provides a more reliable and accurate measure of describing the spatial distribution of two markers. Furthermore, the thresholding of images, which is commonly used to reduce noise in low intensity regions, often introduces bias as it is sensitive to the co-localization method chosen and how it is deployed. By contrast, RWC uses a weighting scheme to penalize low intensity regions in an image, thereby eliminating the need for manual thresholding and effectively reducing a major source of bias in quantification. We have shown that in a completely automated manner, RWC can be used to accurately quantify the spatial-temporal translocation of a cargo molecule as it passes through various organelles in the early secretory pathway. We have also demonstrated the use of RWC in improving clustering and classification of images [2, 3]. In this brief report, we present the implementation of the RWC algorithm in three different image analysis platforms, widely used by cell biology researchers. We believe that these implementations will be a valuable ‘easy-to-use’ resource for co-localization studies by the wider scientific community.

## Implementations

### JACoPx – an ImageJ plugin

ImageJ (http://imagej.nih.gov/ij/index.html) is an open-source, Java-based image analysis program developed at the National Institutes of Health [4]. It works with multiple operating systems (Windows, Mac OS, OS X, Unix-based systems) and its open architecture allows for extensions using custom Java plugins and macros. ImageJ is one of the most widely used image processing systems with applications in biological and medical sciences including analysis of microscopy, pathology and radiology images [5]. JACoP (Just Another Colocalization Plugin) is an ImageJ plugin that provides a variety of co-localization measures including Pearson’s coefficient, Overlap coefficient, Manders’coefficient and Costes’ automated threshold. We have extended JACoP to now include RWC coefficients. This extended plugin (JACoPx) provides an option along with the default measures to additionally calculate RWC coefficients. Since JACoP is already widely used, and provides an extensive collection of co-localization measures, we reasoned that implementing RWC into the same plugin will enable users to use the same familiar tool to also extract RWC coefficients, and thus also be able to easily compare these measures against other coefficients. JACoPx can be easily installed using the jacopx_.jar file provided through our website.

To install JACoPx:

- Download the associate jar file (jacopx_.jar) to the plugins folder within the ImageJ installation directory;
- Restart ImageJ. JACoPx should now be available for use under plugins tab.

### MeasureCorrelationx – a CellProfiler module

CellProfiler (http://cellprofiler.org) is a Python-based, open source high-throughput image analysis system developed at the Broad Institute of MIT and Harvard [6]. CellProfiler is available for Mac OS X, Windows and Linux operating systems. CellProfiler has a modular design enabling users to choose the image processing routines specific to their assays. CellProfiler is a highly ranked cell image analysis tool that provides an interface to build analysis pipelines using the image processing modules as building blocks [7]. CellProfiler is designed for high-throughput analysis where quantitative phenotypic measurements can be extracted from thousands of images automatically. One of the measurement modules available in CellProfiler, called ‘MeasureCorrelation’, provides the Pearson’s co-localization coefficient for a pair of images. We have extended this module to include Overlap, Mander’s coefficient, Costes’ automated threshold and RWC coefficients. This extended module (MeasureCorrelationx) now enable users to extract a variety of co-localization measures with relative ease.

To install MeasureCorrelationx:

- Download the associated Python script (MeasureCorrelationx.py) to a folder;
- Point the “CellProfiler plugins directory” within the preferences option to this folder;
- Restart CellProfiler. MeasureCorrelationx should now be available under the measurement modules list.

### RWC_Co-localization.script – an Acapella script for Columbus

Columbus Image Data Storage and Analysis System (http://www.perkinelmer.com/pages/020/cellularimaging/products/columbus.xhtml) is a proprietary web enabled system for storage and analysis of image data developed by Perkin Elmer. Columbus is a modular system similar to CellProfiler allowing users to build custom analysis pipelines and perform high-throughput analysis of very large image data sets. Modules are developed using Acapella scripts (Evotec Technologies GmbH), and Tony J. Collins (MacBioPhotonics, McMaster University, Canada) and the Andrews Lab (Sunnybrook Research Institute, Toronto, ON, Canada) have provided a suite of co-localization procedures (MBF_ColocalisationCoefficientsb03.proc) written in the Acapella scripting language. We have extended this procedure to create an Acapella script (RWC_Co-localization.script) that implements the RWC algorithm, such that this script can also be used as an independent ‘assay’ within the Columbus system. The script works with 3 channel input images and the user can select the two channels between which the co-localization coefficients wish to be calculated. The script allows for segmentation and detection of cell nuclei and cytoplasmic areas using the various inbuilt detection options, and then extracts co-localization coefficients on the objects identified. This script can be downloaded and directly imported as an assay in a ‘ready-to-use’ format.

## Conclusions

We offer a web resource with implementation of the RWC algorithm in three of the most popular fluorescence microscopy image analysis software systems. While quantitative co-localization is a critical measure used in many conventional cell biology experiments, there is an increasing need for an analytical resource to perform such calculations on large-scale image data sets. We have provided open-source programs containing implementations of the RWC coefficient that can be easily incorporated into existing image analysis pipelines. All the software implementations are accessible via the following website: http://simpsonlab.pbworks.com/w/page/48541482/Bioinformatic_Tools.
